# Characterization of β2-microglobulin expression in different types of breast cancer

**DOI:** 10.1186/1471-2407-14-750

**Published:** 2014-10-07

**Authors:** Kesheng Li, Huifen Du, Xiaowen Lian, Suisheng Yang, Dandan Chai, Chunya Wang, Rong Yang, Xuezhong Chen

**Affiliations:** Department of Medicine Biotechnology, Medicine and Science Research Institute of Gansu Province, Lanzhou, China; Department of Breast Surgery, Tumor Hospital of Gansu Province, Lanzhou, China; Department of Surgery, Tumor Hospital of Gansu Province, Lanzhou, China; Department of Pathology, Tumor Hospital of Gansu Province, Lanzhou, China

**Keywords:** Βeta-2-microglobulin, Molecular subtypes, Breast cancer

## Abstract

**Background:**

Βeta-2-microglobulin (β2-M) has been demonstrated as a growth factor and signaling molecule in breast cancer and leukemia. The purpose of the study is to characterize β2-M expression in molecular subtypes of breast cancer, thereby investigating the mechanism of β2-M action in breast cancer.

**Methods:**

β2-M and B-Cell Lymphoma/Leukemia 2 (Bcl-2) transcript expression levels in breast cancer tissue and the corresponding normal tissue were quantified using real-time PCR. The protein expression levels of β2-M, estrogen receptor (ER), progesterone receptor (PR), human epidermal growth factor receptor 2 (HER-2), tumor protein 53 (p53) and Ki67 were determined by immunohistochemical (IHC) staining. Following silencing of the *β2-M* by siRNA, the levels of Bcl-2, ER, PR and HER-2 transcripts and the protein expression levels in human breast cancer cells were measured by real-time PCR and western blotting, respectively.

**Results:**

The expression of β2-M transcripts demonstrated no significant differences between the four breast cancer molecular subtypes and no significant correlations with age, clinical stage or lymph node metastasis. β2-M transcript expression demonstrated a positive correlation when compared to Bcl-2 transcript expression (P < 0.05). The β2-M protein expression was significantly higher in breast cancer when compared with benign breast tumors (P < 0.01), and have no significant correlation with age, clinical stage or lymph node metastasis. There was a significant difference demonstrated in β2-M protein expression in the four breast cancer molecular subtypes (P < 0.05), and between the ER^+^ and ER^−^ groups (P < 0.01); however, no significant difference was demonstrated between the HER-2^+^ and HER-2^−^ groups. β2-M protein expression had a negative correlation with ER protein expression (P < 0.01), a positive correlation with p53 protein expression (P < 0.01), and no correlation with Ki67 protein expression. *β2-M* silencing significantly inhibited Bcl-2 mRNA expression, but did not inhibit ER, PR and HER-2 mRNA expression in MCF-7 cells (ER^+^, PR^+^ and HER-2^−^). In addition, Bcl-2 and HER-2 mRNA expression were significantly up-regulated in MDA-MB-231 cells (ER^−^, PR^−^ and HER-2^−^), which is consistent with the silencing effect seen at the protein level.

**Conclusions:**

β2-M expression demonstrated a significant difference in the four breast cancer molecular subtypes, and may be related to apoptosis regulation in breast cancer.

## Background

β2-M is a low molecular weight protein and is part of the HLA antigen molecule, representing the invariant light chain [1, 2]. It exists in the membrane of almost all nucleated cells, and is detectable in all body fluids as a shedding product of the cell membrane [3]. In renal disease that presents with the damage of the tubuli renales and increased glomerular filtration rates, quantities of β2-M in urine are increased. If the rate of glomerular filtration is reduced, the level of serum β2-M is increased [4]. Therefore, serum and urine concentrations of β2-M are used to monitor glomerular and tubular nephropathies [5]. The levels of serum and urine β2-M are also found to be increased in patients with some tumors, including solid tumors and leukemia [6–9]. Thus, the levels of serum β2-M have become one of the most important prognostic factors and predictors of survival in patients with some tumors [7–9]. Studies have reported that β2-M is a growth factor and signaling molecule in cancer cells [10–12] and is also a pleiotropic signaling molecule that regulates p21-activated kinases (PAK), androgen receptor, vascular endothelial growth factor (VEGF), fatty acid synthase [12], lipid-raft-mediated growth and survival signaling pathways [13]. The role of β2-M has been demonstrated in several solid cancers and leukemia; however, the mechanism of β2-M action is poorly understood.

Although increased β2-M serum levels in patients with breast cancer have been previously reported [6, 9, 12], the clinical value of β2-M as a prognostic factor and predictor of survival, and its mechanism in patients with breast cancer, need further study, since breast cancer has different molecular subtypes [14] and patients with the same clinical stages and pathological types of breast cancer, treated with same scheme, have different therapeutic and prognostic effects. The aim of this study is to characterize β2-M expression in the different breast cancer molecular subtypes, thereby investigating whether β2-M is involved with apoptosis regulation in breast cancer. The results of this study will be useful in confirming β2-M-mediated signaling as a new target for breast cancer therapy.

## Methods

### Tissue samples

The tissue samples in this study were collected from 330 patients with breast cancer and 123 patients with benign breast tumors, identified by clinical and histopathological evidence, who underwent surgery at Tumor Hospital of Gansu province. The 164 breast cancer and 123 benign breast tumor tissue samples were from formalin-fixed, paraffin-embedded (FFPE) tissue specimens obtained from the pathology department during surgery performed between 2011 and 2012. The other 166 breast cancer and adjacent normal fresh tissue samples were obtained from surgical specimens resected from patients without previous chemotherapy and radiotherapy during operations performed or examinations by centesis between 2010 and 2013. The fresh tissue samples were frozen at −80°C for preparation of total RNA extraction. The clinicopathological information, including age, node status and tumor-node-metastasis (TNM), was obtained from each patient’s clinical and pathologic reports. The patient characterizations are listed in Table 1. The Medical Ethics Committee of Medicine and Science Research Institute of Gansu Province approved the study protocol (Reference number: A201301310001) and all patients gave consent for participating in the study and publishing the study results.

**Table 1:** **Patient characterization**

Variable	IHC staining	Real-time PCR and IHC	Total
**Case no.**	164	166	330
**Age (years)**			
<59	126	143	269
≥60	37	21	58
**Stage**			
I + II	55	57	112
III + IV	29	40	69
**Lymph node metastasis**			
Present	59	34	93
Absent	63	53	116
**Benign breast tumors**	123	0	123

### Total RNA extraction and real-time PCR

Total RNA was extracted from the tumor tissues and the adjacent normal tissues using Trizol reagent (Shenggong Biotechnology, Shanghai, China) according to the manufacturer’s instructions. The cDNA was synthesized by reverse transcription using the RNA as a template and reverse transcriptase (Shenggong Biotechnology), according to the manufacturer’s recommendations. The SYBR premix Ex TaqTM (TaKaRa Biotechnology, Dalian, China) was used for the real-time PCR. Briefly, the 20 μl reaction contained 10 μl of SYBR premix Ex TaqTM, 1 μl of DNA template, 0.4 μl of each primer and 8.2 μl dH_2_O. The PCR cycling conditions included the following steps: 37°C for 5 min, 95°C for 30 s, and 40 cycles of 95°C for 5 s to 60°C for 30 s. β-actin mRNA was used as the internal control and the reaction mix without template DNA was used as the negative control. All of the samples were measured 3 times independently, and the resulting fluorescence curves represent the number of DNA copies expressed. The primers are listed in Table 2.

**Table 2:** **Primers used in the real-time PCR**

Gene	Forward primer	Reverse primer
***β-actin***	5′-TGGCACCCAGCA	5′-CTAAGTCATAGT
	CAATGAA-3′	CCGCCTAGAAGCA-3′
***β2-M***	5′-CGGGCATTCCTG	5′-GGATGGATGAAA
	AAGCTGA-3′	CCCAGACACATAG-3′
***Bcl-2***	5′-TGTATGAACTGA	5′-CACCTGGCAGCG
	GCAATGTGCAAGA-3′	TAGGGTAA-3′

### IHC staining

Expression of β2-M, ER, PR, HER-2, p53 and Ki67 in tumor tissues were detected by IHC staining. Sections from the surgical specimens fixed in 10% formalin and embedded in paraffin were used for IHC staining by the standard method. Briefly, the paraffin-embedded tissues were cut to a 3-mm thickness, de-paraffinized with xylene and rehydrated through graded ethanol washes. The sections were autoclaved in 10 mM citrate buffer (pH 6.0) at 120°C for 5 min for antigen retrieval, cooled to 26°C, treated with 3% H_2_O_2_ for 5–10 min to block endogenous peroxidase activity, and then washed with phosphate-buffered saline (PBS, pH 7.3) for 3 min 3 times. After being blocked with 10% normal calf serum in PBS for 10–15 min, the sections were incubated at 37°C for 2–3 h with anti-β2-M (developed by Department of Medicine Biotechnology, Medicine and Science Institute of Gansu Province, Lanzhou, China) at a 1:1000 dilution, then washed with PBS. Next, the sections were incubated with biotinylated secondary antibody (ZYMED, South San Francisco, CA, USA) for 10–15 min at 37°C, and then washed with PBS. Horseradish peroxidase polymer conjugate (SP-9000 Histostain TM-plus Kit, ZYMED) was then applied to the sections at 37°C for 10–15 min followed by PBS washes. Finally, the sections were incubated with 3-3′-Diaminobenzidine (DBA) for 5–10 min. The nuclei were lightly counterstained with hematoxylin. A negative control was run simultaneously by omitting the primary antibody. The stained slides were independently assessed by two pathologists, and any differences in decision outcomes were resolved by consensus. To evaluate the expression of β2-M, the tissue sections were examined under a microscope at a magnification of 200×. The results of the tissue sections staining were estimated according to the following: positive, cytoplasm staining or both cytoplasm and cytomembrane staining (mainly cytoplasm staining); negative, no cytomembrane staining. The intensity of staining was classified according to the following scale: negative, 0; weak, 1; moderate, 2; and strong, 3. Staining was semi-quantitatively scored according to proportion of stained cells by the following scale: 0, no cells stained; 1, <10%; 2, 10-50%; and 3, >50% of cells stained. The staining intensity scores and proportion of stained cells were added; the cut-off value for positive expression of β2-M was defined as moderate staining with >10% of cells stained.

The ER, PR, HER-2, p53 and Ki67 (Beijing Zhongshan Jinqiao, Beijing, China) staining procedure was same as that of β2-M described above. The results of tissue sections stained for ER, PR, p53 and Ki67 were determined according to the following: positive, nucleus staining or both nucleus and cytoplasm staining (mainly nucleus staining); negative, only cytoplasm staining. The cut-off value for positive expression was the same as that of β2-M. The results of tissue sections stained for HER-2 were scored by the ASCO/CAP system: positive, cytomembrane staining; negative, no cytomembrane staining. The cut-off value for positive expression of HER-2 was defined as having more than 10% of the cells stained.

### Silencing of the *β2-M*gene by siRNA in breast cancer cells

Three siRNAs targeting different regions of β2-M mRNA [GenBank: NM014002] were designed and purchased from GenePharma (Suzhou, China). Scrambled siRNA (GenePharma) that does not target any gene was used as the negative siRNA control. All siRNAs are detailed in Table 3. Breast cancer cells (MCF-7 and MDA-MB-231, purchased from the Shanghai Cell Bank of Chinese Academy of Sciences, Shanghai, China) were transfected with the siRNAs using Lipofectamine-2000 according to the manufacturer′s instructions. Briefly, cells were seeded in a 6-well-plate at a density of 1 × 10^5^ cells/well with antibiotic-free medium 12 h before transfection. Two microliters of each siRNA (40 μM) were mixed with 3 μl Lipofectamine-2000 in 50 μl serum-free RPMI-1640 medium and were allowed to incubate at room temperature for 25 min to form a complex. After washing cells with PBS, the 50 μl transfection mixtures were added to each well with 1950 μl RPMI-1640 medium containing 10% FBS at a final concentration of 40 nM siRNA. Forty-eight hours after transfection, the cells were collected for real-time RT-PCR and western blotting.

**Table 3:** **β2-M siRNAs used in this study**

siRNAs	Sequences
**Negative control (NC)**	5′-UUCUCCGAACGUGUCACGUTT
	TTAAGAGGCUUGCACAGUGCA-5′
**siR-1 (si1)**	5′-CUCCAAAGAUUCAGGUUUATT
	TTGAGGUUUCUAAGUCCAAAU-5′
**siR-2 (si2)**	5′-CCGACAUUGAAGUUGACUUTT
	TTGGCUGUAACUUCAACUGAA-5′
**siR-3 (si3)**	5′-CACAGCCCAAGAUAGUUAATT
	TTGUGUCGGGUUCUAUCAAUU-5′

### Western blotting

The cultured cells were washed twice with ice-cold PBS and lysed on ice in lysis buffer containing protease and phosphatase inhibitor cocktails. Following a 5-min incubation, the cell lysate was collected by centrifugation at 4°C for 10 min at 12,000 rpm. Twenty micrograms of total protein was separated by SDS-PAGE. The protein was transferred to a nitrocellulose membrane, blocked and then probed with appropriate antibodies. The protein was visualized using horseradish peroxidase-conjugated secondary antibodies and the FluorChem FC2 imaging system. Anti-β2-M antibody (self-government), anti-β-actin antibody (Rockland, Gilbertsville, PA, USA), anti-Bcl-2 antibody, anti-HER-2 antibody, anti-ER antibody, anti-PR antibody and horseradish peroxidase-conjugated secondary antibody (Invitrogen, Carlsbad, CA, USA) were used for western blotting.

### Statistical analysis

The data were analyzed using the Statistical Package for Sciences software (IBM-SPSS version 22). The statistical significance of intergroup differences was evaluated using a χ^2^ test. P < 0.05 was considered statistically significant.

## Results

### Expression of β2-M transcripts in breast cancer tissues

The expression of β2-M transcripts was detected by real-time PCR in all 166 breast cancer specimens and their paired normal tissues. The specimens were divided into three groups according to the ratio of the β2-M transcript levels in the tumor tissue (T) to that in the normal tissue (N): up-regulation (T/N > 2), down-regulation (T/N < 0.5) and no change in expression (2 > T/N > 0.5). The results are shown in Table 4 and demonstrate the following: 15.66% (26/166) up-regulation, 20.48% (34/166) down-regulation and 63.86% (106/166) no change in expression of β2-M transcripts was observed in the breast cancer specimens. In addition, no significant correlations were found between β2-M transcript expression level and age, clinical stage or lymph node metastasis; 18.67% (31/166) up-regulation, 28.92% (48/166) down-regulation and 52.41% (87/166) no change in expression of Bcl-2 transcripts were observed in the breast cancer specimens, and no significant correlations were found between Bcl-2 transcript expression level and age, clinical stage or lymph node metastasis.

**Table 4:** **Expression of β2-M transcript and clinicopathological and molecular type parameters in breast cancer specimens**

Variable	β2-M			P-value	Bcl-2			P-value
	T/N	T/N	T/N		T/N	T/N	T/N	
	>2	2-0.5	<0.5		>2	2-0.5	<0.5	
**Case no.**	26	106	34		31	87	48	
**Stage**								
I-II	11	35	11	0.67	8	37	12	0.483
III-IV	5	27	8		11	19	10	
**Age (years)**								
<40	3	15	4	0.739	4	13	5	0.907
40-60	21	74	26		24	61	36	
>60	2	16	3		3	11	7	
**Lymph node metastasis**								
Present	3	22	9	0.316	5	17	12	0.981
Absent	10	34	9		7	27	19	
**Molecular subtype**								
Luminal A	4	33	12	0.928	12	28	9	0.282
Luminal B	8	30	11		7	23	19	
Overexpression of HER-2	5	19	6		6	15	9	
Basal-like	2	8	3		3	4	6	
**ER** ^**+**^	12	63	23	0.731	19	51	28	0.840
**ER** ^**−**^	8	29	10		10	22	15	
**HER-2** ^**+**^	14	50	18	0.426	14	40	28	0.336
**HER-2** ^**−**^	6	42	15		15	33	15	

### Expression of β2-M transcripts in different breast cancer molecular subtypes

The expression levels of the β2-M transcripts in different breast cancer molecular subtypes are shown in Table 4. The expression levels of the β2-M transcripts, including up-regulation, down-regulation and no change, demonstrated that there was no significant difference in the four breast cancer molecular subtypes (P = 0.928). No significant difference was observed in β2-M transcript expression levels between the ER^+^ and ER^−^ breast cancer groups (P = 0.731) and the HER-2^+^ and HER-2^−^ breast cancer groups (P = 0.426). There was no significant difference in the expression levels of Bcl-2 transcripts in the four breast cancer molecular subtypes, including up-regulation, down-regulation and no change (P = 0.282). In addition, no significant difference was observed in Bcl-2 transcript expression between the ER^+^ and ER^−^ breast cancer groups (P = 0.840) and HER-2^+^ and HER-2^−^ breast cancer groups (P = 0.336) (Table 4).

### Association of β2-M transcript expression with Bcl-2 transcript expression

The association of β2-M transcript expression with Bcl-2 transcript expression is shown in Table 5. The β2-M transcript expression levels have a positive correlation with Bcl-2 transcript expression levels (P = 0.011).

**Table 5:** **Association of β2-M transcript expression with Bcl-2 transcript expression**

β2-M transcript expression	Bcl-2 transcript expression				P-value
	Up-regulation	No difference	Down-regulation	Total no.	
**Up-regulation**	20	14	6	40	0.011
**No difference**	31	68	21	120	
**Down-regulation**	8	12	10	30	
**Total no.**	59	94	37	190	

### Expression of β2-M protein in the breast cancer tissues

**Figure 1:**
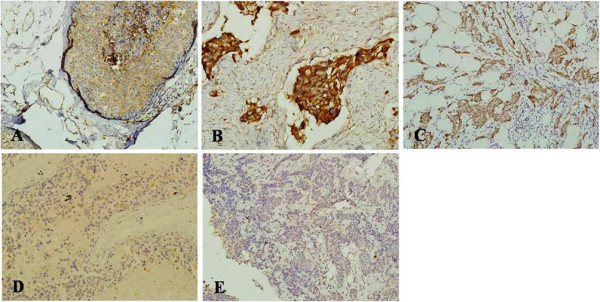
**β2-M IHC staining in breast cancer tissue. A)** Strong cytomembrane staining; **B)** Strong cytoplasm staining; **C)** Moderate cytoplasm staining; **D)** Weak cytoplasm staining; **E)** Negative staining. The tissue sections were examined under a microscope at a magnification of 200×.

The paraffin-embedded sections from the 164 patients’ specimens with breast cancer and the 123 patients with benign breast tumors were immunohistochemically stained using β2-M, ER, PR, HER-2, p53 and Ki67 antibodies (Figure 1). The results demonstrate that 67.68% (111/164) of sections from breast cancer patients were positively stained by the β2-M antibody, which is significantly higher than the 34.14% (42/123) positive staining from patients with benign breast tumors (P < 0.01). No significant correlations were found between β2-M protein expression and age, clinical stage or lymph node metastasis. In addition, 37.03% p53 and 88.40% Ki67 positive staining were found in the breast cancer patient sections, and no significant correlations were found between p53 protein expression and age, clinical stage or lymph node metastasis. The expression of the Ki67 protein had a significant correlation with lymph node metastasis (P < 0.01), but no significant correlation with age or clinical stage (Table 6).

**Table 6:** **Expression of β2-M protein in different breast cancer molecular types**

Variable	β2-M		p53		Ki67	
	Positive staining	P-value	Positive staining	P-value	Positive staining	P-value
	(%)		(%)		(%)	
**Breast cancer**	67.68 (111/164)	<0.01				
**Benign breast tumor**	34.14 (42/123)					
**Age (years)**						
<59	65.87 (83/126)	0.617	38.83 (40/103)	0.506	90.56 (96/106)	0.130
>60	70.27 (26/ 37)		32.15 (10/31)		80.64 (25/31)	
**Stage**						
I + II	61.81 (34/55)	0.515	25.53 (12/47)	0.227	82.97 (39/47)	0.136
III + IV	68.96 (20/29)		34.78 (8/23)		95.65 (22/23)	
**Lymph node metastasis**						
Present	67.79 (40/59)	0.956	40.00 (18/45)	0.279	97.82 (45/46)	<0.01
Absent	68.25 (43/63)		29.62 (16/54)		79.62 (43/54)	
**Molecular subtype**						
Luminal A	56.25 (36/64)	0.034	25.00 (16/64)	<0.01	77.41 (48/62)	<0.01
Luminal B	53.33 (16/30)		32.00 (8/25)		96.15 (25/26)	
Overexpression of HER-2	84.61 (22/26)		70.83 (17/24)		100.00 (26/26)	
Basal-like	73.68 (14/19)		38.88 (7/18)		94.44 (17/18)	
**ER** ^**+**^	58.06 (54/93)	<0.01	27.05 (23/85)	<0.01	82.75 (72/87)	<0.01
**ER** ^**−**^	81.39 (35/43)		56.09 (23/41)		100.00 (43/43)	
**HER-2** ^**+**^	72.41 (42/58)	0.180	50.98 (26/51)	<0.01	98.11 (52/53)	<0.01
**HER-2** ^**−**^	61.62 (53/86)		28.04 (23/82)		83.13 (69/83)	

### Expression of β2-M protein in different breast cancer molecular subtypes

The β2-M protein expression levels in different breast cancer molecular subtypes are shown in Table 6. The expression levels of β2-M protein demonstrate significant differences in the four breast cancer molecular subtypes (P = 0.034); the rate of positive staining was 56.25% (Luminal A), 53.33% (Luminal B), 84.61% (Overexpression of HER-2) and 73.68% (Basal-like). Significant differences were also observed in the β2-M protein expression levels between the ER^+^ and ER^−^ breast cancer groups (P < 0.01); 58.09% (ER^+^) and 81.39% (ER^-^) positive staining were observed in the breast cancer specimen sections. There was no significant difference in the β2-M protein expression levels between HER-2^+^ and HER-2^−^ breast cancer groups (P = 0.180). Both p53 and Ki67 proteins demonstrated significant differences in expression between the four molecular subtypes (P < 0.01), between the ER^+^ and ER^−^ groups (P < 0.01), and between HER-2^+^ and HER-2^−^ breast cancer groups (P < 0.01).

### Association of β2-M protein expression with ER, p53 and Ki67 protein expression

The association of β2-M protein expression level with ER, p53 and Ki67 protein expression levels is shown in Table 7. The level of β2-M protein expression has a negative correlation with ER protein expression (P < 0.01), a positive correlation with p53 protein expression (P < 0.01) and no correlation with Ki67 protein expression (P = 0.6).

**Table 7:** **Association of β2-M protein expression with ER, p53 and Ki67 protein expression**

Variable	β2-M protein expression			P-value
	Positive (+)	Negative (−)	Total no.	
**Case no.**	85	56	141	<0.01
**ER** ^**+**^	46	47	93	
**ER** ^**−**^	39	9	48	
**Case no.**	84	52	136	<0.01
**p53** ^**+**^	36	13	49	
**p53** ^**−**^	48	39	87	
**Case no.**	81	52	133	0.60
**Ki 67** ^**+**^	73	46	119	
**Ki 67** ^**−**^	8	6	14	

### Silencing of *β2-M*gene by pre-designed siRNAs

**Figure 2:**
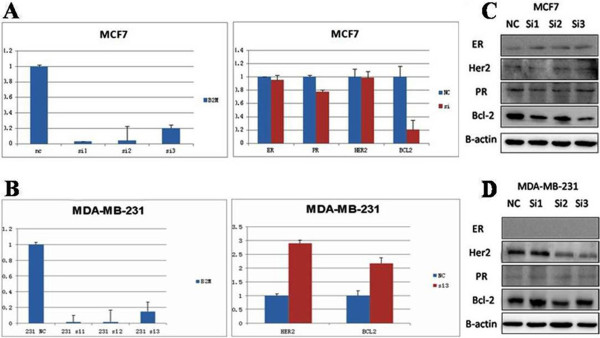
**Silencing of the** ***β2-M*** **gene by siRNAs in breast cancer cells. A)** β2-M siRNAs significantly inhibited the Bcl-2 mRNA expression, but did not inhibit the ER, PR, and HER-2 mRNA expression in MCF-7 cells (ER^+^, PR^+^ and HER-2^−^); **B)** β2-M siRNAs significantly up-regulated the Bcl-2 and HER-2 mRNA expression in MDA-MB-231 cells (ER^−^, PR^−^ and HER-2^−^); **C)** ER, HER-2, PR and Bcl-2 protein levels in MCF-7 cells, with or without β2-M siRNAs; **D)** ER, HER-2, PR and Bcl-2 protein levels in MDA-MB-231 cells, with or without β2-M siRNAs.

Silencing effects of the pre-designed β2-M siRNAs were examined in MCF-7 and MDA-MB-231 cells, and a scrambled siRNA was used as the negative control. All three siRNAs showed a significant silencing effect (P < 0.01) and knocked down 80 to 98% of the β2-M mRNA in comparison with the scrambled siRNA (Figure 2). Among the β2-M siRNAs tested, only the siR-3 siRNA showed a significant effect on downstream genes and therefore this siRNA was selected for silencing of the *β2-M* gene. The mRNA transcript and protein expression levels of β2-M, ER, PR, HER-2 and Bcl-2 were detected by real-time PCR and western blotting after *β2-M* silencing in MCF-7 and MDA-MB-231 cells. As shown in Figure 2, siR-3 significantly inhibited Bcl-2 mRNA expression, but did not inhibit the levels of ER, PR, and HER-2 mRNA expression in MCF-7 cells, which is consistent with the silencing effect at the protein level. The Bcl-2 and HER-2 mRNAs were significantly up-regulated by siR-3 silencing in MDA-MB-231 cells, which is also consistent with the silencing effect at the protein level (Figure 2).

## Discussion

Overexpression of β2-M has been observed in patients with breast cancer [6, 9], and studies have shown that β2-M supports breast cancer bone metastasis [15]. In this study, our results show that: (1) the expression of β2-M transcripts only demonstrate a 15.66% up-regulation in the breast cancer specimens, no significant difference in the four breast cancer molecular subtypes, and no significant correlations with age, clinical stage or lymph node metastasis. β2-M transcript expression has a positive correlation with Bcl-2 transcript expression. (2) Overexpression of the β2-M protein was significantly higher in breast cancer tissues compared to that in benign breast tumors, and has no significant correlation with age, clinical stage or lymph node metastasis in breast cancer. Expression levels of the β2-M protein are significantly different in the four breast cancer molecular subtypes, significant differences were demonstrated between the ER^+^ and ER^−^ breast cancer groups, but not between the HER-2^+^ and HER-2^−^ breast cancer groups. (3) β2-M protein expression has a negative correlation with ER protein expression, a positive correlation with p53 protein expression and no correlation with Ki67 protein expression. (4) β2-M siRNAs have different silencing effects in the different breast cancer molecular subtypes; it significantly inhibited Bcl-2 mRNA expression and did not inhibit the ER, PR and HER-2 mRNA expression in MCF-7 cells (ER^+^, PR^+^ and HER-2^−^); however, there was significant up-regulation in the Bcl-2 and HER-2 mRNA expression levels in MDA-MB-231 cells (ER^−^, PR^−^ and HER-2^−^), which is also consistent with the silencing effect at the protein level.

In conclusion, the aforementioned results demonstrate the following. First, the expression levels of the β2-M transcript show no significant difference between different breast cancer molecular subtypes, and no significant association with age, clinical stage or lymph node metastasis. Second, the expression level of the β2-M protein was significantly up-regulated by upstream genes or factors in breast cancer; its expression has different regulation pathways in the different breast cancer molecular subtypes, and has a negative correlation with ER protein expression. Therefore, the expression of the β2-M protein may be regulated by other signaling pathways beside the ER signaling pathway; the mechanism of this regulation needs to be further defined. Third, β2-M transcript expression has a positive correlation with Bcl-2 transcript expression. Consequently, the overexpression of β2-M transcripts may cause up-regulation of the Bcl-2 transcripts in breast cancer, and restrain apoptosis in breast cancer cells. Bcl-2 is a target protein of the ER genome singling pathway in breast cancer cells (MCF-7), and modulates apoptosis in breast cancer cells [16]. ERα is a key molecule of ER singling pathway [17, 18], and estrogen can markedly promote the proliferation of breast cancer cells with ERα overexpression [19]. Therefore, β2-M may promote proliferation and restrain apoptosis in breast cancer cells through the ER genome singling pathway in breast cancer with ER overexpression. Fourth, β2-M siRNAs significantly inhibited Bcl-2 mRNA expression, but did not inhibit ER, PR and HER-2 mRNA expression in breast cancer cells with ER^+^, PR^+^ and HER-2^−^ status. In contrast, there was significant up-regulation in Bcl-2 and HER-2 mRNA expression levels in breast cancer cells with ER^−^, PR^−^ and HER-2^−^ status. The different breast cancer molecular subtypes are caused by different pathologies [20], are regulated by different singling pathways, and β2-M may have different functions in the different breast cancer molecular subtypes. Other studies have shown that β2-M is a signaling and growth-promoting factor for human renal cell carcinoma and prostate cancer bone metastasis. Interrupting the β2-M signaling pathway may induce apoptosis in tumor cells and β2-M may stimulate growth and improve osteocalcin (OC) and bone sialoprotein (BSP) gene expression in human prostate cancer cells via activating cyclic AMP (cAMP)-dependent PAK signaling pathway [10, 21]. Human β2-M monoclonal antibodies may have the effect of inducing apoptosis *in vitro*, and have therapeutic effects in mouse models of myeloma and other hematological tumor cells. The monoclonal antibodies may induce apoptosis and accomplish therapeutic functions by activating the c-Jun N-terminal kinase (JNK) and the caspase-9-dependent cascade, inhibiting PI3K (Phosphatidylinositol-3 kinase)/Akt and ERK (extracellular signal-regulated kinase) [13]. β2-M may accelerate human renal cell carcinoma cell growth via activation of PI3K/Akt and ERK, and induce phosphorylation of the Bcl-xL/Bcl-2-associated death promoter (Bad). The β2-M antibody may induce the human renal cell carcinoma cells apoptosis by inhibiting the phosphorylation of Akt and ERK, and activating JNK, resulting in the phosphorylation of Bcl-2 and decreased phosphorylation of Bad, leading to apoptosis [22]. Thereby, we deduced that β2-M may resist apoptosis by activating PI3K/Akt and ERK. Moreover, the β2-M siRNAs inhibited Bcl-2 mRNA expression by inhibiting the phosphorylation of Akt and ERK in breast cancer cells with ER overexpression. HER-2 may increase the antiapoptotic proteins survivin and Bcl-2 via activating the ERK and PI3K signaling pathways [23]. Accordingly, β2-M may promote apoptosis by inhibiting HER-2 expression, resulting in inhibition of PI3K/Akt and ERK signaling pathways. In addition, β2-M siRNAs may up-regulate the Bcl-2 mRNA expression via increasing HER-2 expression in breast cancer cells with ER^−^, PR^−^ and HER-2^−^ status. However, the regulation of the β2-M signaling pathways in the different breast cancer molecular subtypes need further study.

Briefly, the results of this study indicate that expression of β2-M is significant differences in four breast cancer molecular subtypes, which may lead to different functions of apoptosis regulation in breast cancer. These results will also be useful to understanding β2-M signaling pathways regulation, and help to identify new targets for the treatment of breast cancer patients.

## Conclusions

The expression of β2-M is significantly different in four breast cancer molecular subtypes, and the β2-M siRNAs have different silencing effects in the different breast cancer molecular subtypes. β2-M may be involved in apoptosis regulation of breast cancer, and understanding the regulation of the β2-M signaling pathways will help to identify new targets for the treatment of patients with breast cancer.

## Abbreviations

β2-M: Βeta-2-microglobulin
Bcl-2: B-Cell Lymphoma/Leukemia 2
ER: Estrogen receptor
PR: Progesterone receptor
HER-2: Human epidermal growth factor receptor 2
p53: Tumor protein 53
PAK: p21-activated kinases
VEGF: Vascular endothelial growth factor
FFPE: Formalin-fixed, paraffin-embedded
TNM: Tumor-node-metastasis
IHC: Immunohistochemical
PBS: Phosphate-buffered saline
DBA: 3-3′-Diaminobenzidine
T: Tumor tissue
N: Normal tissue
OC: Osteocalcin
BSP: Bone sialoprotein
cAMP: Cyclic AMP
JNK: c-Jun N-terminal kinase
PI3K: Phosphatidylinositol-3 kinase
ERK: Extracellular signal-regulated kinase
Bad: Bcl-xL/Bcl-2-associated death promoter.
